# Ergonomics of an In‐Patient Hospitalization

**DOI:** 10.1111/hex.70261

**Published:** 2025-04-10

**Authors:** Amy Louise Gilliland

**Affiliations:** ^1^ School of Human Ecology University of Wisconsin‐Madison Madison Wisconsin USA

**Keywords:** autism, ergonomics, health economics, in‐patient, medical care, neurodivergence, patient work

## Abstract

This article documents inpatient work, including activities, number of nurses, shift changes and other staff encounters for the first time. In 2022, the researcher (patient) had a traumatic limb injury and began three 6–8 day stays in three different hospitals. Nursing care was provided by 37 different nurses and 23 certified nursing assistants in 50 different shifts over 21 days. There were 35 other unique encounters by other medical care staff. As an experienced qualitative researcher, the patient relied on field observation skills. Thirty‐eight distinct inpatient activities were grouped into five general categories: Logistics, Arrangements, Self‐care, Healing and Social Management. This article reveals that patients inside the hospital actively engage in various tasks to navigate and manage their health‐related goals. These responsibilities may be overwhelming for the patient, who is without resources to solve their own problems.

**Patient Contribution:**

This paper is 100% from the patient's perspective and their original contribution.

## 1 Introduction

My career has been primarily in healthcare education and research. As a birth doula and patient advocate with poorly understood medical disorders, I have developed skills to navigate the medical system. In October 2022, I experienced a traumatic limb injury. I was surprised and burdened by the amount of work expected of me as an inpatient. As a trained field observer and qualitative researcher, I applied my skills to analyse and document expected and unexpected inpatient tasks.

## 2 What Is Patient Work?

The term ‘patient work’ was first used in 1993 by sociologist Anselm Strauss to refer to the ‘exertion of effort and investment of time on the part of patients or family members to produce or accomplish something’ [1]. ‘Patient ergonomics’ refers to studying and improving patients' efforts or work in accomplishing their health goals. A review of 67 publications reveals that most patient work is done alone, in private, and often imposes cognitive burdens on patients, especially those with low amounts of support [2]. Further, patient work is strongly influenced by contextual factors. Patient tasks were categorized by whether the task was visible to others; whether it involves cognitive skills; if the task must be conducted collaboratively with others or can be done alone; and if the task was done with the purpose of creating more resources. Their main conclusion was that ‘health‐related work for patients is ever present, invisible and overwhelming’.

Understanding how health care experiences are integrated into everyday life reveals how patients not only manage their condition, but manage health care information [3]. Managing information is a separate task from managing one's condition. Some information can be categorized as helpful or useful, i.e. making a procedure less painful. Other information management adds additional patient work, such as how to obtain a prior authorization for a procedure. Together, condition management and information management can be overwhelming. My accident and extensive notetaking provided an opportunity to document inpatient work and information management tasks. My observations are grounded in interactions with 37 different nurses and 23 certified nursing assistants over 50 different shifts over the 21 days.

## 3 Researcher Background

As a qualitative researcher, I have published using grounded theory and hermeneutic phenomenology. I had multiple qualifications: a research fellow at a large university working on projects related to doula support, nurse–patient interactions and the interpersonal neurobiology of the brain – including how trauma is encoded and decoded. Second, I have degrees in interpersonal communication and certifications in human sexuality education and doula training. I provided professional labour support for over 30 years as a birth doula. At the time of the accident, I was a 60‐year‐old female with a poorly understood and rare genetic disorder. Because of that, I have spent decades negotiating and advocating with providers.

## 4 Setting

Six weeks before the hospitalization, I read Vincent Montori's book, *Why We Revolt* about the effect of industrialized health care on systems and workers [4]. Because of my own chronic illness, I became fascinated by the concept of ‘invisible patient work’. While away from home, I fell down a flight of stairs which broke my leg and crushed my ankle. The injury required several surgeries, hospital observation during recovery, and occupational and physical therapy. Due to limitations beyond our control, only one intimate family member was able to assist me and that was only for an hour each day. During my initial week of hospitalization, I became aware of the lack of continuity with nursing staff. At Hospital A, I was a patient in the orthopaedic trauma ward. At Hospital B, I was a patient in the rehabilitation hospital wing. At Hospital C, I was in an ‘overflow’ ward with patients hospitalized for different reasons. There was a month's break between Hospital B and Hospital C.

## 5 My Work as a Patient

Upon discharge from Hospital C, I had 25 full pages of notes on a yellow pad. I had also kept a meticulous diary of my sleep, symptoms, medications, bodily functions and phone calls. As I began to detail everything I had been responsible for during my hospital stays, I ended up with a list of 40 tasks that could be grouped into five categories: Logistics, Arrangements, Self‐care, Healing and Social Management (see Table 1). Logistics tasks required extensive investments of time, energy or complex problem‐solving to accomplish. Arrangements were simpler problems that only had to be solved once, but that didn't mean they were easy to solve. Self‐care tasks are part of the ever‐present work I have to do as a patient with a rare genetic disorder. They would happen no matter what my circumstances. Healing tasks were related specifically to my injury. Social Management tasks required conscious brain engagement, awareness and sufficient brain glucose to do well. All of these tasks feel necessary for my survival in each hospital atmosphere.

**Table 1:** Types of inpatient work.

Task distinguishing feature	Logistics (repeated tasks, lengthy)	Arrangements (problem solving)	Self‐care (ever‐present, ongoing)	Healing (related to injury)	Social management (required intensive brain engagement and mindfulness)
Tasks	Negotiating with insurance companies (i.e. coverage, authorization) talking with insurance company patient advocate Negotiating with the discharge coordinator(s) Ordering meals daily on phone or tablet Additional food	Obtaining over‐the‐counter medications and care items (Ordered and delivered) Clothing to dress myself and fit over my cast for two climates (Ordered and delivered) Laundry while in hospital Post‐discharge wheelchair Disability accommodations (hotel) post‐discharge Car and air travel to another state Place to live while living in wheelchair Medical care after discharge, both local and distant Additional aftercare planning	Daily/weekly meal planning (Limited diet, can't repeat foods, nutrition) Managing symptoms and muscle cramps (stretching, exercises, etc.) Daily physical cares, such as bathing, brushing teeth, and toileting Managing sensory environment of hospital Problem‐solving in the moment Emotion management Pain management Meditation	Seeking support from the rehab therapist Managing my acute trauma symptoms Managing sleep and sleeping Resting and healing from my injury Getting around with a wheelchair and a walker Daily physical and occupational therapy Meetings with care team Adapting to schedule of hospital	Reassuring my family over the phone Negotiating relationships with CNA's (i.e. Getting towels so that I could shower on my own) Negotiating ongoing power struggles between the therapy staff and the nursing staff Approaching the charge nurse when receiving bad care Working with insurance advocate Managing roommates, their habits and visitors Managing visitors and well wishes Adjusting to a new nurse every four to 12 h Asking questions and finding answers

*Note:* Lists of tasks by one inpatient during three 6–8‐day hospital stays in three different facilities.

Table 2 shows average activities in every 24 h period. Because of the short amount of sleep and interrupted sleep, I felt chronically sleep deprived. Table 3 lists an average daily schedule, which remained fairly consistent across facilities.

**Table 2:** Average time spent in patient work activities (24 h cycle).

Activity	Time spent
Sleeping, resting, meditating (eyes closed)	8.0 h
Physical therapy/occupational therapy	3.5 h
Logistics, arrangements	4.0 h
Self‐care tasks	3.0 h
Healing tasks	2.0 h
Social management tasks	3.5 h

**Table 3:** Average Day in Hospital.

Time of day	Activity
5:00–5:30	RN visit Morning Medication, awakened if sleeping
5:30–6:00	Try to get back to sleep
6:00–6:30	Staff shift change, new nurse introduction
6:30–7:00	Try to get back to sleep
7:00–8:00	Breakfast arrives, morning care tasks; order meals for next day
8:00–9:00	Physical therapy I
9:00–9:30	Break
9:30–10:30	Occupational therapy
10:30–12:00	Break, shower, dress, nap, RN visit with medications; discharge coordinator visits; phone calls; possible nurse staff change
12:00–13:00	Lunch, midday care tasks, phone, rest
13:00–14:00	Physical Therapy II
14:00–15:00	Trauma Therapy or Patient Advocate Phone Visit or Occupational Therapy II; Phone calls
15:00–16:00	Nap
16:00–17:00	My Visitor! Clean laundry, extra food, sundries, supplies, pep talk
17:00–18:00	Dinner, rest, evening meds, shift change with new nurse introduction
18:00–20:00	Roommate has visitors; evening physical therapy and self‐care tasks
20:00–21:00	Phone logistics, prepare for sleep
21:00–23:00	Sleep
23:00–23:30	Awakened for night medications and visit; possible nurse staff change
23:30–2:00	Sleep
2:00–2:30	Middle of the night care tasks
2:30–5:00	Sleep

Looking more deeply, Logistics involved negotiating with my insurance companies over coverage and authorizations for care. This was separate from the Patient Advocate. A retired ICU and trauma nurse, the Patient Advocate gave me insight into what I was experiencing as a patient. I could not have navigated the hospital stay without them. Since I did not have any knowledgeable family members, I discussed medical decisions with my Advocate. They gave me the correct terminology to ask for things with insurance company representatives and the Discharge Coordinators regarding coverage.

Responsibilities varied from facility to facility, which was somewhat confusing. In Hospital A, it was the senior physical therapist who discussed my rehabilitation options and provided the discharge paperwork. In Hospital B, there was an assigned discharge coordinator, but they couldn't coordinate anything out of state. They gave me a list of what to do. This meant I had to locate my own wheelchair, transportation, insurance authorizations and locate a new doctor and clinic in the area where I went to recover.

In addition, my condition means I require a special diet of rotating foods and preparations, which was hard to maintain on the hospital menu. Hospitals A and B required that only three meals could be ordered at one time and they could only be ordered over the phone. This took me a half hour or more each day. At Hospital C, I could order from a tablet; however, the menu was difficult to navigate. It had hidden rules that didn't appear until you violated them (i.e. no more than two condiments).

Arrangements are the kinds of tasks that are invisible or simple to accomplish when not in a hospital bed. Getting my prescribed medications was challenging, and I went without my daily medications for over half a week. I relied heavily on ordering items online and delivery services. I had no clothing compatible with my injuries and no access to laundry. I was often rescued by the Therapy staff to resolve these daily dilemmas. For example, an Occupational Therapist took me to use the washer and dryer, using the reason they were helping me learn how from my wheelchair.

Self‐care tasks are ones that I would have had to do no matter what, such as rotating foods, doing my everyday physical therapy and exercises, and managing the sensory environment of the hospitals. Accomplishing self‐care in the hospital was incredibly challenging. The ambient noise, especially in Hospital B, was constant and overwhelming. I wore soundproof ear protection intended for construction sites to cope. Those 8 days were something I had to get through. I had to learn a whole new way of thinking, moving and accomplishing daily living. I felt frightened, overwhelmed, in pain, immobilized and responsible for my accident and its consequences. Emotion management was absolutely necessary to function but difficult to do without a reliable support system. In Hospital B, where I was on the rehabilitation floor, the occupational therapists, physical therapists, patient advocates and trauma therapists were absolutely necessary for my survival. They were also instrumental to the Healing tasks which were directly related to my injury, such as adapting to the hospital's routine and schedules, managing my acute trauma symptoms, managing sleep, getting around with a walker and wheelchair, meeting with the care team, daily physical and occupational therapy, trauma therapy and resting my body.

The most significant task I had as an inpatient was the work of social relationships. I was dependent on another person to receive my medication, to receive permission to move or toilet when needed, to offer me information that could improve the quality of my hospital stay or my life, and to do logistics work on my behalf. Even though it is someone's job, the quality of the relationship with a patient does, in part, determine the quality of that patient's care. I received care from 37 different nurses and 23 certified nursing assistants over 50 different shifts over the 21 days. In the three hospital stays combined, I interacted three or more times with two outpatient coordinators (6 visits), eight physical therapists or assistants (24 visits), four occupational therapists (16 visits) and one trauma therapist (3 visits). There were also 35 other singular encounters with emergency transport workers, hospital transport workers, testing professionals and specialty physician teams.

In addition, I talked on the phone for 30–90 min a day during my stays in Hospitals A and B with my Patient Advocate, depending on what the current issue was or problems to be solved. Once I left Hospital B and the state, we were required to sever contact.

## 6 Hospital Environments

All the hospitals were noisy. Hospital A had closed doors; Hospital B required open doors; at Hospital C, whoever walked out the door left it open or shut. I wasn't asked. I purchased soundproof headphones at Hospital B to cope with the continuous ambient noise. Most rooms had a window with a pleasant view; however, my bed wasn't placed, so I could see out of it. I didn't watch television or stream media; I didn't want any more stimulation. The most significant part of the environment was the people I had to interact with. Unlike the nursing staff, the certified nursing assistants (CNA) often had the same patients.

## 7 Establishing Relationships and Power Struggles

In Hospital B, I experienced power struggles with several certified nursing assistants (CNA). It became very clear to me whether a CNA liked me or not. The CNAs I encountered liked their patients to be independent but not too independent. Occupational therapy cleared me to toilet and shower on my own. If I waited until after 10 AM in the morning, there would be hot water. This meant I would need towels at a nontraditional time. CNAs who liked me would bring me towels without asking. Other CNAs would respond to my request as if I was being self‐centred or just not bring me towels. There was one CNA who said she would write me up as an uncooperative patient by not bathing at her preferred time. This same CNA would take any towels I had stashed the night before in my dresser because I was ‘hoarding’ them and not bring new ones. I told my physical therapist and was told to get linens from the PT closet from now on.

Another power struggle that I found myself in the middle of occurred between the Occupational Therapy (OT) and Physical Therapy (PT) staff and the Nursing staff. To treat my chronic condition, PT cleared me to exercise in the late afternoon and before bed in the physical therapy room. Unfortunately, nobody told me that this had never happened before or that there was a history of patients escaping from the unit. My activity of wheeling around the unit independently in the early evening made the nursing staff very nervous. I discovered on my last night they were watching me on the security cameras waiting for me to try and leave the unit. They didn't understand why I was going into the PT room at night. I got the distinct feeling that the nursing staff thought the PT staff gave me this privilege to irritate them, while the PT staff thought that the nursing staff was shortsighted. I felt that I landed right in the middle of an ongoing conflict that really had nothing to do with me. Yet I had to negotiate this territory to have a good experience as a patient.

## 8 Conclusion

Much of patient work takes place in a border zone between formal healthcare and self‐care. To benefit from formal care, patients must do a lot of complicated work on their own requiring skills, resources and initiative [5]. A person's social situation, especially their employment, influences whether patient work gets done or not. Lastly, patient work is not formally recognized as part of the organization of care in general by practitioners or by patients; it's implied rather than stated outright and obviously. This means the patient has to figure out what their work is rather than receiving support from a health care provider or system. Other patients are likely to be as surprised as I was by the amount of work or kind of work they must accomplish. By documenting invisible patient work, we can make this study more valued and seen by healthcare providers and integrate it into pre‐existing patient care models. To achieve patient‐ or person‐centred care, we have to include the unique and privileged knowledge from patients in the clinical information space [6]. Clinicians, third‐party payers and healthcare economists need the information about patient work to make patient‐centred and coordinated treatment planning decisions.

## Author Contributions

**Amy L. Gilliland:** conceptualization, investigation, writing – original draft, methodology, writing – review and editing, visualization, validation, formal analysis, resources.

## Conflicts of Interest

The author declares no conflicts of interest.

## Data Availability

The data that supports the findings in this article are included in the support materials. Due to the sensitive nature of healthcare data, it may be shared upon reasonable request.

## References

[1] A. Strauss , Continual Permutations of Action (Aldine De Gruyter, 1993).

[2] K. Yin , J. Jung , E. Coiera , et al., “Patient Work and Their Contexts: Scoping Review,” Journal of Medical Internet Research 22, no. 6 (2020): e16656, 10.2196/16656. 32484449 PMC7298639

[3] C. A. Smith and P. R. Masters , “College Students and Patient Work: Health Information Management by Emerging Young Adults,” Library & Information Science Research 45, no. 1 (2023): 101216, 10.1016/j.lisr.2022.101216.

[4] V. Montori , Why We Revolt: A Patient Revolution for Careful and Kind Care (Mayo Clinic Press, 2020).

[5] Sá Rogvi , A. D. Guassora , N. Tvistholm , G. Wind , and U. Christensen , “'It Is a Full‐Time Job to be Ill': Patient Work Involved in Attending Formal Diabetes Care Among Socially Vulnerable Danish Type 2 Diabetes Patients,” Qualitative Health Research 31, no. 14 (2021): 2629–2640, 10.1177/10497323211041590. 34612745

[6] E. L. Papautsky and E. S. Patterson , “Patients Are Knowledge Workers in the Clinical Information Space,” Applied Clinical Informatics 12, no. 01 (2021): 133–140, 10.1055/s-0041-1723022. 33626585 PMC7904384

